# Excess of Fat in the Artificial Feeding of Infants

**Published:** 1905-01-28

**Authors:** 


### EXCESS OF FAT IN THE ARTIFICIAL FEEDING OF INFANTS.

A COMMON fault in the artificial feeding of babies, and one which is attended with serious effects, is to give an insufficient amount of fat. Several of the advertised infants' foods are deficient in this constituent, and many of the brands of condensed milk which find favour with poor mothers who cannot nurse their babies are prepared from separated milk and in consequence are very markedly deficient in fat. This defect and its results have received a great deal of attention from the medical profession during the last few years ; but hitherto little has been said of any drawbacks following the use of a diet which contains too much fat. Emmett Holt, an acknowledged authority on the feeding of infants, affirms that very serious disturbances of digestion may result from an excess of fat in the food. A child who is taking too much fat will in all probability thrive exceedingly at first, but in the course of a few months it is likely to lose its appetite and to show signs of gastric and intestinal indigestion. Among these unwelcome manifestations may be regurgitation of food or habitual vomiting, wasting, constipation, convulsions, and tetany. Once the true cause of these disturbances is recognised, the remedy is obvious. An aperient should be given, and the food must be limited in quantity and well diluted with water until the gastro-intestinal irritation has subsided. Subsequently care should be taken that the food does not contain an undue proportion of fat, not a difficult matter, seeing that the excess is more often due to design than accident. A common source of the error, according to Dr. Holt, is the use of rich Jersey or Alderney milk and cream in preference to that of other breeds, whose milk contains a smaller percentage of fat.

